# Propagation of fluid-driven aseismic slip fronts under upper crustal conditions

**DOI:** 10.1126/sciadv.aeb0234

**Published:** 2026-07-08

**Authors:** François Passelègue, Nicolas Brantut, Hervé Chauris, Pierre Dublanchet

**Affiliations:** ^1^ Université Côte d’Azur, CNRS, Observatoire de la Côte d’Azur, IRD, Géoazur, Sophia Antipolis, France.; ^2^ Department of Earth Sciences, University College London, London, UK.; ^3^ GFZ Helmholtz Centre for Geosciences, Potsdam, Germany.; ^4^ Mines Paris, PSL University, STIM, Fontainebleau, France.; ^5^ Mines Paris, PSL University, Centre for Geosciences and Geoengineering, Fontainebleau, France.

## Abstract

Fracture mechanics models suggest that aseismic slip may either lag behind or outpace the fluid pressure front, depending on injection conditions and the fault’s initial stress state. However, direct experimental validation of these predictions has been lacking. Here, we report laboratory experiments on granite samples under upper crustal stress conditions, where we simultaneously tracked the propagation of fluid pressure and aseismic slip during fluid injection. We show that aseismic slip lags the pressure front at low injection rates and low initial stress but rapidly outpaces it under high-rate injection or near-failure initial stress conditions. The transition is governed by a dimensionless loading parameter that integrates injection rate, fault strength, and initial shear stress. Our results provide direct support for fracture mechanics models of fluid-induced aseismic slip and suggest that in critically stressed crustal faults, rupture propagation may commonly outpace fluid diffusion.

## Teaser

Aseismic slip triggered by fluid injection can outrun pressure diffusion depending on stress and injection rate.

## INTRODUCTION

Understanding the propagation of fluid-driven frictional ruptures is crucial for assessing the hazard of fluid-induced seismicity, whether arising from natural processes (*1*, *2*) or human activities (*3*–*6*). These ruptures are often inferred from seismicity along natural faults (*7*–*11*) or near anthropogenic fluid injection sites (*3*, *12*–*14*). In cases of induced seismicity, it is well established that the injected fluid volume primarily controls the cumulative aseismic moment (*15*) and can influence the maximum magnitude of induced earthquakes (*16*, *17*).

Seismological observations indicate that seismicity migration often follows a square-root dependence on time, consistent with fluid diffusion along a fault plane. However, this pattern is not universal; in some cases, seismicity propagates linearly with time, suggesting alternative mechanisms such as stress transfer or aseismic slip (*14*, *18*). Moreover, hydraulic diffusivity estimates derived from seismicity migration are typically one to two orders of magnitude larger than those observed in laboratory experiments and natural rock formations (*19*, *20*). This discrepancy may be explained by the fact that, under certain conditions, seismicity is linked to a fluid-driven aseismic slip front, *R*, that also grows as $\sqrt{t}$ (*21*, *22*). The existence of propagating aseismic slip results in a complex and nonsystematic relationship between seismicity patterns and the propagation of the fluid pressure front, *L*. The interplay between the slip and fluid fronts introduces substantial variability in seismic responses, complicating efforts to generalize the spatiotemporal evolution of induced seismicity.

A key question remains: Under what conditions is induced seismicity primarily governed by the propagation of the fluid pressure front, *L*, versus an aseismic slip front, *R*, which may be driven by *L* but can either lag behind or outpace it? Recent theoretical studies suggest that the transition between these two end-member cases is governed by a loading parameter, *T*, which depends on the initial stress state of the fault and the magnitude of the pore pressure change (*21*–*23*). However, experimental validation or field observations to test this hypothesis remain lacking. On the one hand, the absence of continuous, in situ measurements of active fault deformation and hydrological processes limits our understanding of fluid-activated aseismic slip, which currently relies on indirect observations such as seismicity migration (*2*, *10*, *11*) or surface geodetic data (*1*). On the other hand, measuring both *R* and *L* along a limited-sized experimental fault remains challenging (*24*–*26*), as it does during in situ fluid injection experiments along shallow aseismic faults (*27*, *28*), where estimates of the initial state of stress are also lacking. Determining the key parameters controlling the growth of injection-induced ruptures is critical for improving predictive models of induced seismicity, particularly in applications such as geothermal energy production, wastewater disposal, and carbon sequestration.

Here, we address this question by conducting laboratory experiments where the fluid pressure front, *L*, and the aseismic slip front, *R*, can be tracked directly under controlled stress conditions. Our experiments suggest that the question of whether fluid-induced aseismic slip lags behind or outpaces fluid migration is governed primarily by the loading parameter identified in earlier theoretical work (*21*, *22*), suitably modified to account for the injection scenario.

## RESULTS

The experimental fault consists of a saw-cut granite specimen, oriented at an angle θ of 30° with respect to the principal stress $\sigma_{1}$. Fluid injection was carried out under varying stress conditions through a borehole that directly connects a high-pressure fluid reservoir to the fault plane at its top edge. A second borehole, located on the opposite side of the fault, was used to monitor pore pressure changes (Fig. 1A). In addition, three individual pore pressure sensors were positioned at different locations along the fault to track the spatiotemporal evolution of fluid pressure during injection (*29*, *30*).

**Fig. 1.**
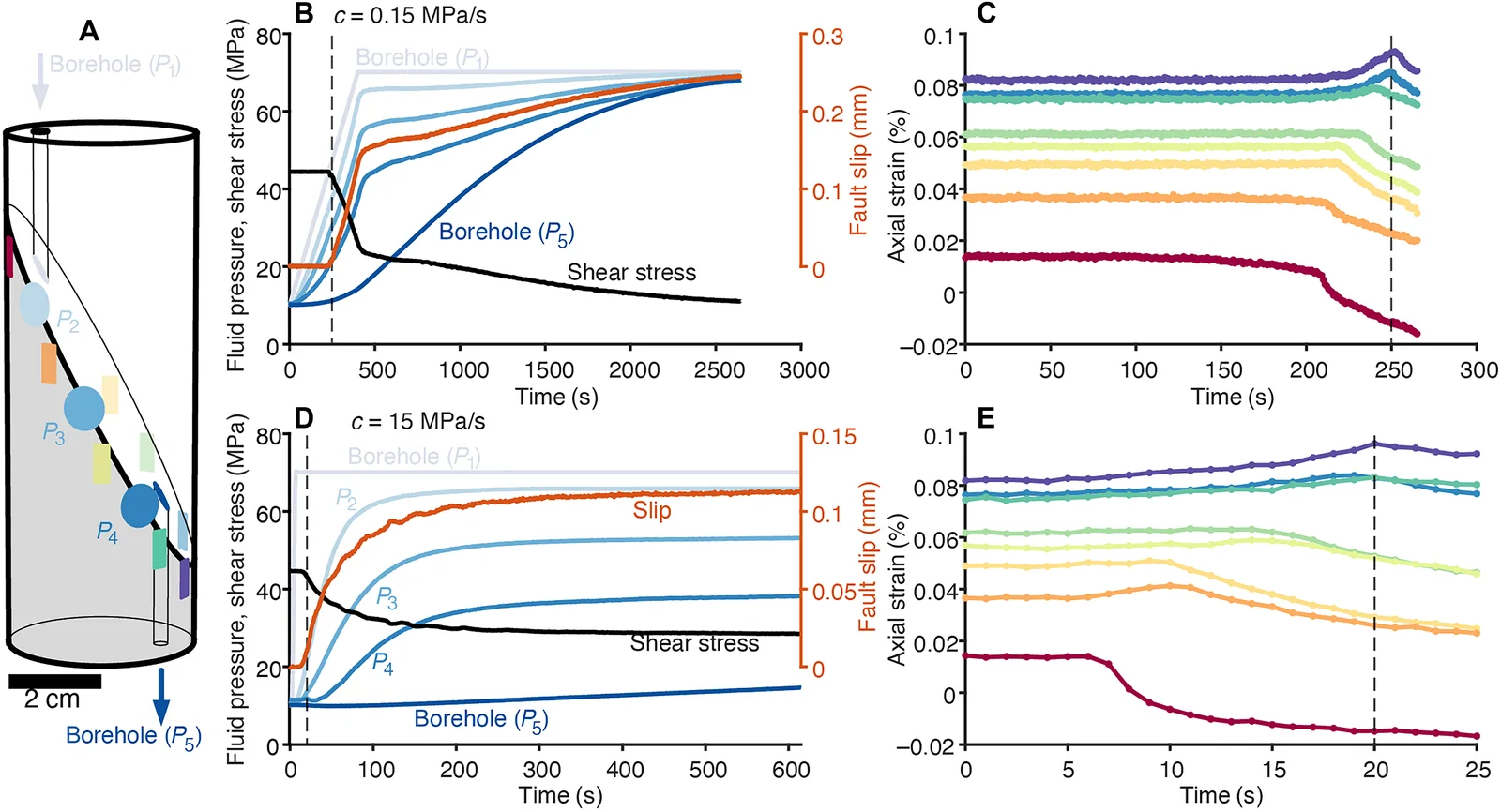
Experimental methods. (**A**) Schematic representation of the sample assembly used in this study. The host rock is Westerly Granite. Rectangles indicate the location of strain gauges around the fault. The injection borehole and the measurement borehole correspond to pressures $P_{1}$ and $P_{5}$, respectively. (**B**) Evolution of macroscopic shear stress, macroscopic fault slip, and fluid pressure measured at five different locations [colors correspond to those shown in (A)] during an injection performed at $\tau_{0}/\tau_{p}=0.6$, with a constant pressure rate of *c* = 0.15 MPa/s. (**C**) Evolution of axial strain recorded by the strain gauge array before the onset of macroscopic slip, indicated by the dashed line. The strain data are organized by distance from the injection site. Colors match those of the markers shown in (A). (**D**) Same as (B), but for the injection conducted at $\tau_{0}/\tau_{p}=0.6$ with a constant pressure rate of *c* = 15 MPa/s. (**E**) Same as (C), but for the injection conducted at $\tau_{0}/\tau_{p}=0.6$ with a constant pressure rate of *c* = 15 MPa/s.

To complement fluid pressure measurements, an array of eight strain gauges was placed around the fault to monitor changes in axial strain (Fig. 1A). This array was used to detect the location of the aseismic slip front during fluid injection (*31*).

The experiments were carried out under stress relaxation conditions (*20*, *24*) (see Materials and Methods) at an initial (Terzaghi) effective confining pressure of $\sigma_{3}^{\prime }=70$ MPa. The background confining pressure and the initial fluid pressure were set to $\sigma_{3}=80$ MPa and $P_{f}=10$ MPa, respectively. Before injection, we probed the frictional strength $\tau_{p}$ of the fault by loading the sample at constant rate until slip was detected. In a first stage, the differential stress ($\sigma_{1}^{\prime }-\sigma_{3}^{\prime }$) was increased to reach a value of initial shear stress $\tau_{0}=\left(\sigma_{1}^{\prime }-\sigma_{3}^{\prime }\right)\text{sin}\left(2\theta \right)/2$ equal to 60% of the fault strength (fig. S1). Injections were then carried out at constant pressure rates *c* of 0.15, 1.5, and 15 MPa/s, until the complete release of differential stress. In a second stage, the differential stress was raised to 90% of the fault strength. Under these conditions, four different constant pressure rates *c* were tested: 0.015, 0.15, 1.5, and 15 MPa/s (see table S1). Note that the fluid pressure rates were imposed constant up to a maximum value of 70 MPa ($\sigma_{3}^{\prime }=10$ MPa). If fault reactivation did not occur at this target value, then the fluid pressure was kept constant until the complete release of differential stress. This case only occured for the maximum injection rate tested (*c* = 15 MPa/s).

We present typical mechanical data recorded at $\tau_{0}/\tau_{p}=0.6$ for injection rates of *c* = 0.15 and 15 MPa/s in Fig. 1. At low injection rates, the fluid pressure increase at the injection site leads to a gradual rise in fluid pressure detected across all pore pressure sensors before the onset of macroscopic slip (Fig. 1B). At any given time, the pressure measured at each sensor decreases with increasing distance from the injection site, indicating the propagation of a fluid pressure “front” along the fault. The evolution of axial strain recorded by the strain gauges reveals that strain release initiates earlier near the injection site and later at locations farther along the fault (Fig. 1C), indicating the occurrence of slip propagation. Once strain release initiates on all strain gauges, macroscopic fault slip is recorded by the external displacement transducer, and a drop in shear stress is measured with the external load cell. This reduction in shear stress mirrors the evolution of slip along the fault up to the end of the injection phase (Fig. 1B), in agreement with the stress relaxation condition imposed by the fixed piston position.

At higher injection rates, only the sensor that is closest to the injection site records an increase in fluid pressure before slip onset (Fig. 1D). In addition, deviations from linearity on the strain gauges propagate faster than at *c* = 0.15 MPa/s (Fig. 1E). Once the macroscopic slip begins, the release of stress also mirrors of the slip occurring along the fault, as observed at low injection rates.

### Inversion of the slip front velocity and of the fluid pressure front

To further analyze the interaction between the slip front and the fluid pressure front, we performed two inversion procedures using the strain gauge and the fluid pressure sensor arrays distributed around the fault (Fig. 1A). First, for each injection, we manually identified the first abrupt release of strain on each strain gauge located around the fault (Fig. 1, C and E). This strain release is expected to indicate the passage of the slip front across the strain gauges, as suggested by synthetic crack propagation using the Green’s functions (*32*) of our fault system (see Materials and Methods and fig. S2). Here, the slip front corresponds to the location along the fault where the strain gauges record the onset of slip. This definition allows us to track the spatial propagation of slip along the fault during injection. Next, we performed an inversion to determine the best solution for the slip front velocity ($\dot{R}$), the nucleation location, and the propagation initiation time, assuming a simplified circular front propagation. The minimization of the difference between the experimental and theoretical arrival times provided the optimal solution for these three parameters. In each injection, the slip front nucleates in the area surrounding the injection site (fig. S3). Increasing injection rate leads to an increase in rupture front velocity (fig. S3). In addition, a high background stress level also seems to enhance $\dot{R}$. For a similar pressurization rate, the slip front propagates faster at $\tau_{0}/\tau_{p}=0.9$ than at $\tau_{0}/\tau_{p}=0.6$. In all experiments presented here, slip propagation remains aseismic, as the observed slip front never attains seismic rupture velocities.

Second, we performed an inversion to reconstruct the fluid pressure distribution along the fault during each injection. To account for dead-volume effects in the pore pressure system, we constrained the inversion by imposing the fluid pressure at the injection site ($P_{1}$) and the pore pressure measured in the second borehole ($P_{5}$). We then inverted for hydraulic diffusivity in both time and space, assuming three independent regions, each monitored by an individual fluid pressure transducer ($P_{2}$, $P_{3}$, and $P_{4}$). The minimization process yielded the optimal solution for the spatiotemporal evolution of fluid pressure during each injection (fig. S4). To track the propagation of the fluid pressure perturbation along the fault, we defined the fluid pressure front as the location where the pressure exceeds a threshold of 1 MPa above the initial uniform fluid pressure along the fault (10 MPa). Although the pressure field evolves diffusively and therefore does not exhibit a sharp front, this threshold provides a consistent operational marker of the spatial extent of the pressure perturbation and allows comparison with the propagation of the slip front. The interaction between the advancing slip front and the fluid pressure front is presented in Fig. 2 for two distinct injection scenarios conducted at 60% of the fault’s peak strength, with injection rates of 0.15 and 15 MPa/s.

**Fig. 2.**
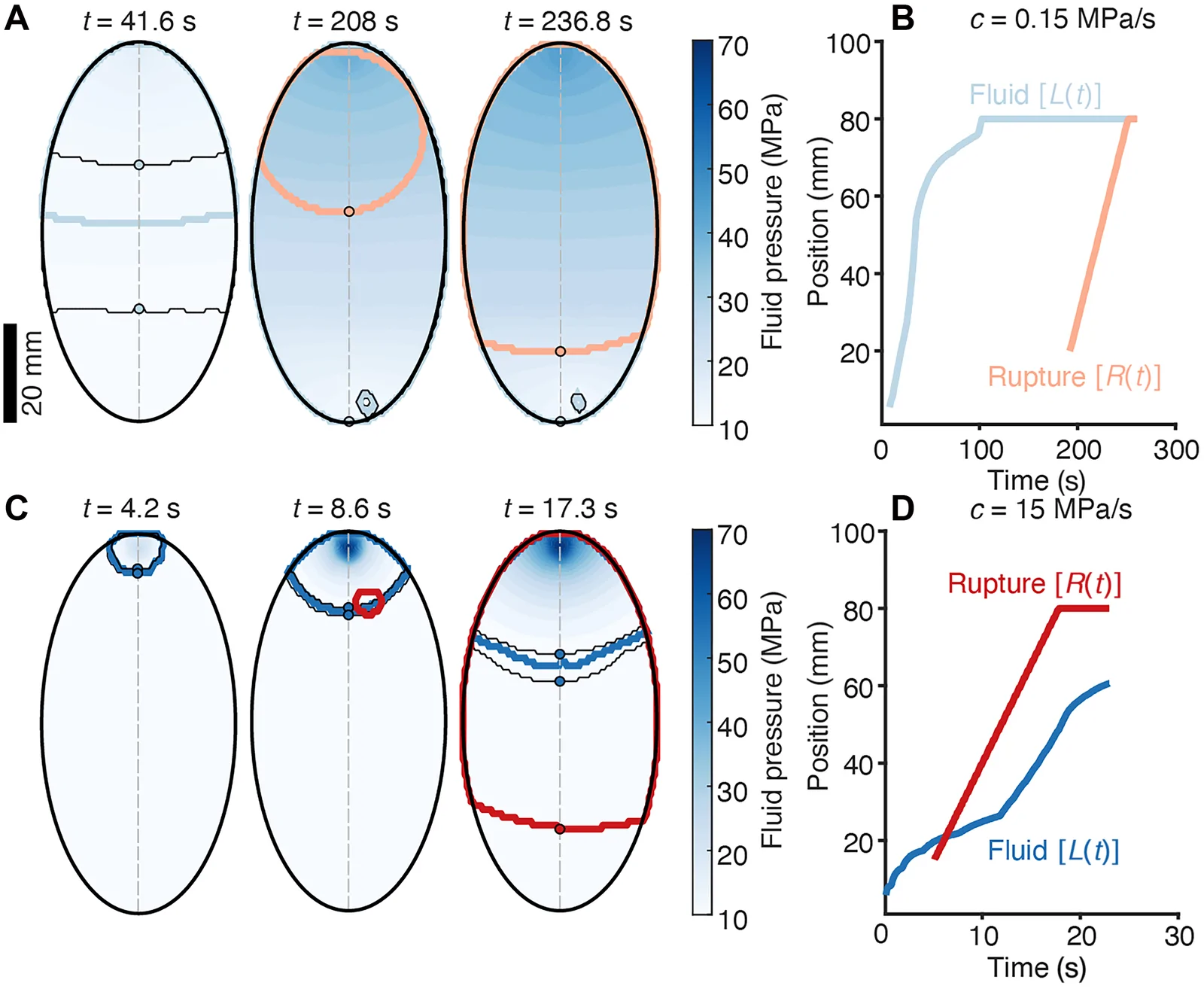
Inversion of *R* and *L* during fluid injection. (**A**) Snapshots at three different times illustrating the propagation of the fluid pressure front *L* (blue) and the slip front *R* (reddish line) during an injection performed at $\tau_{0}/\tau_{p}=0.6$ with a constant pressure rate *c* = 0.15 MPa/s. The color scale represents fluid pressure (same as in Fig. 3). Thin black contours indicate the position of the fluid pressure front *L* for thresholds of 10.7 and 11.3 MPa, while the thick blue contour corresponds to 11 MPa. Colored markers show the positions of the slip front *R* (reddish colors) and the pressure front *L* (bluish colors) along the fault trace (dashed line). (**B**) Temporal evolution of the front positions *R*(*t*) and *L*(*t*) along the fault for the same injection conditions ($\tau_{0}/\tau_{p}=0.6$, *c* = 0.15 MPa/s). (**C**) Same as (A), but for an injection with a higher constant pressure rate *c* = 15 MPa/s. (**D**) Same as (B), but for *c* = 15 MPa/s.

At the lower injection rate (Fig. 2, A and B), the fluid pressure front propagates across the entire fault before the initiation of slip front propagation. Once initiated, the slip front advances at a relatively slow velocity of about 0.6 mm/s and eventually catches up with the fluid pressure front, doing so ~244 s after the onset of injection. This occurs because the spatial extent of the pressure front is limited by the finite size of the experimental fault. At the higher injection rate (Fig. 2, C and D), the fluid pressure front also propagates first. However, at the onset of slip front propagation, *L* remains localized near the injection site. At this stage, *R* propagates at a faster velocity than *L* ($\dot{R}=5$ mm/s), and overtakes it at *t* = 8.56 s (Fig. 2B). These results confirm that an increase in the injection rate facilitates the transition between the two end-member cases: (i) a slip front *R*(*t*) propagating behind the fluid pressure front *L*(*t*) and (ii) a slip front *R*(*t*) initially lagging behind the fluid pressure front *L*(*t*) but eventually outpacing it, propagating ahead of it in the later stages (Fig. 2B).

## DISCUSSION

We now compare the temporal evolution of both the slip front, *R*(*t*), and the fluid pressure front, *L*(*t*), along the long axis of the experimental elliptical fault (Fig. 2) for (i) the two initial stress states tested ($\tau_{0}/\tau_{p}=0.6$ and $\tau_{0}/\tau_{p}=0.9$, respectively) and (ii) each applied pressure rate *c* ranging from 0.015 to 15 MPa/s. Our experimental results indicate that, regardless of the initial stress state, increasing *c* accelerates the propagation of both the fluid pressure front and the slip front (Fig. 3). However, the initial stress state influences the transition between the two end-member cases previously discussed. At $\tau_{0}/\tau_{p}=0.6$, only the injection conducted at *c* = 15 MPa/s allows *R*(*t*) to outpace *L*(*t*), at *t* ≈ 6 (Fig. 3A). In contrast, at $\tau_{0}/\tau_{p}=0.9$, *R*(*t*) outgrowths *L*(*t*) for injections conducted at both *c* = 1.5 MPa/s and *c* = 15 MPa/s, at *t* ≈ 26 s and at *t* ≈ 5 s, respectively (Fig. 3B).

**Fig. 3.**
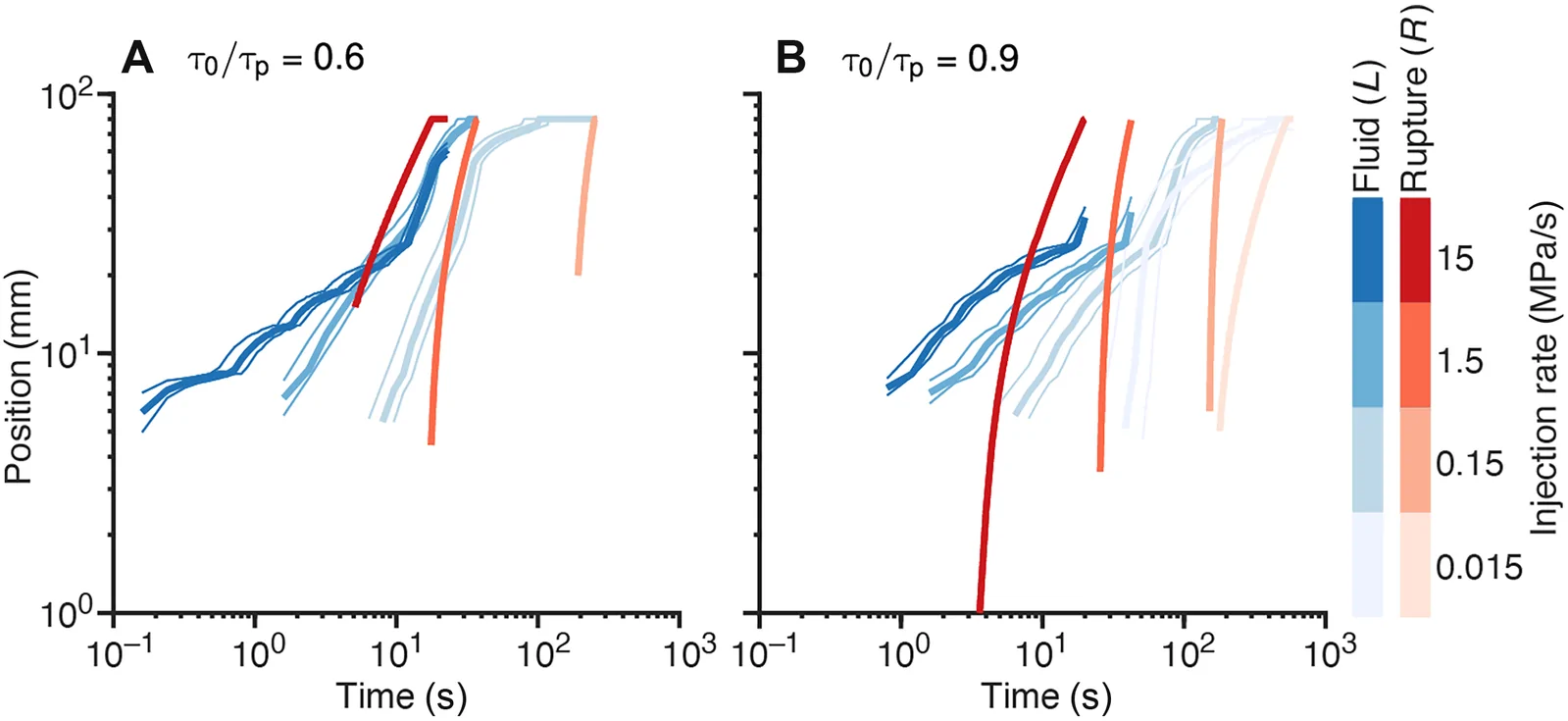
One-dimensional propagation of rupture *R*(*t*) and fluid pressure *L*(*t*). (**A**) Evolution of the rupture dimension (reddish colors) and fluid front position (bluish colors) along the fault length (Fig. 2) as a function of time for injections conducted at $\tau_{0}/\tau_{p}=0.6$. *L*(*t*) represents the location of the fluid front for a threshold of 1 MPa above the initial, uniform pressure field (solid lines), ±0.3 MPa (thin lines). The reddish and bluish color scales correspond to the values of pressurization rate *c* used in each injection. (**B**) Same as (A), but for injections conducted at $\tau_{0}/\tau_{p}=0.9$.

These observations are broadly consistent with theoretical expectations, as both $\tau_{0}$ and *c* are expected to contribute to variations in the loading parameter (*21*, *22*), defined as

$$T=\frac{1-\tau_{0}/\left(f\sigma_{0}^{\prime }\right)}{\Delta p^{*}/\sigma_{0}^{\prime }}$$ (1)

where *f* is the static friction of the fault, $\sigma_{0}^{\prime }$ is the initial effective normal stress along the fault, and $\Delta p^{*}$ represents a characteristic pressure perturbation at the injection site. In our experiments, the appropriate characteristic pressure is $\Delta p^{*}={\mathrm{ca}}^{2}/\alpha$, where *a* is the injection borehole radius and α is the fault hydraulic diffusivity (see Materials and Methods). Equation 1 highlights that an increase in both $\tau_{0}$ or *c*, individually or together, leads to a decrease in *T*, which is expected to control the transition from the case R≪L, termed “marginally pressurized fault,” to the case R≫L, termed “critically stressed fault” (*21*, *22*, *33*, *34*).

In our experiments, there is some uncertainty in the quantification of *T*, since we only have an imperfect knowledge of the friction coefficient, which is assumed constant in the theory of (*22*). In practice, *f* is not constant but depends on slip rate and past history of the fault “state” (*35*, *36*). Here, we estimate *T* using either a constant friction coefficient equal to 0.67, obtained independently on the basis of measurements of $\tau_{p}$, or equal to the normalized time $t\times \alpha /a^{2}$ at the initiation of rupture (see Materials and Methods, Eq. 22). We also use our measurements of *R* and *L* to determine the time history of the amplification factor defined by λ=R/L. In contrast with injection scenarios at constant injected volume rate, where λ is fully determined by *T* and both *R* and *L* grow as $\sqrt{t}$ (*21*, *22*, *34*), we anticipate that λ depends on both *T* and time in the case of injection at constant pressurization rate (see Materials and Methods and fig. S5).

We observe that experiments conducted at large *T* (low stress or low injection rate) tend to produce ruptures that start late and remain initially behind the fluid migration, whereas at low *T*, the rupture rapidly outpaces the fluid (Fig. 4A), in qualitative agreement with model results. The slip front typically nucleates within the pressurized area (fig. S3), so that the initial values of λ are smaller than 1. With ongoing time, λ increases, which indicates that the slip front propagates faster than the pressure front. For the lowest values of *T*, λ eventually surpasses 1. Our results highlight that λ becomes larger than 1 at shorter timescale with decreasing the value of *T*, in agreement with numerical results (fig. S5). These results suggest that the larger the injection rate and the initial shear stress along the fault, the faster *R* will outgrow *L*.

**Fig. 4.**
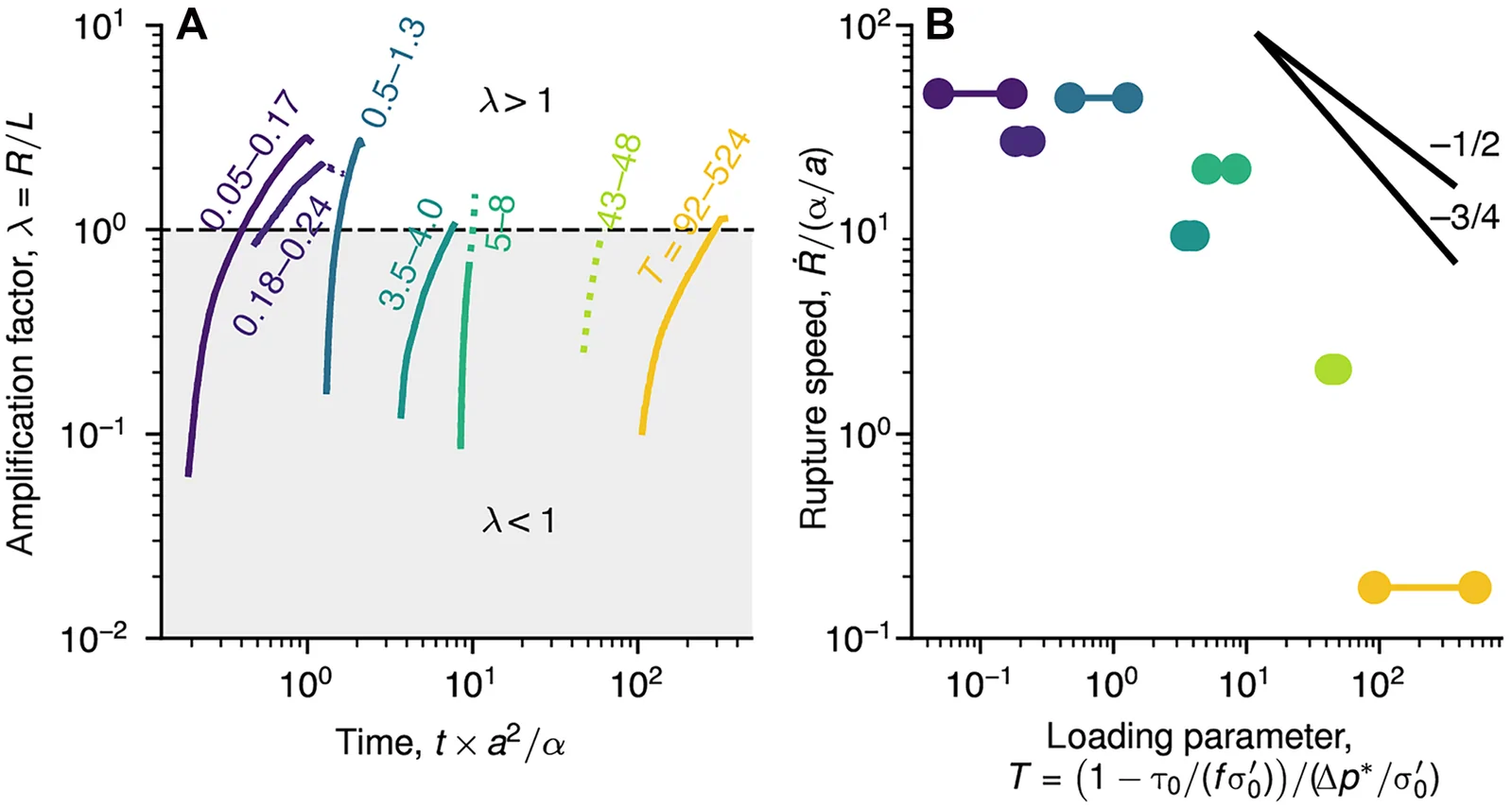
Influence of the loading parameter *T* on slip and fluid propagation. (**A**) Ratio λ=R(t)/L(t) as a function of scaled time for all injections. The colored solid lines show the evolution of λ during the period when both the slip front *R*(*t*) and the fluid pressure front *L*(*t*) are propagating along the fault. The colored dashed lines represent that the evolution of λ after either the fluid pressure front *L*(*t*) or the slip front *R*(*t*) has reached the bottom edge of the fault. The black dashed line corresponds to λ = 1 and marks the transition between the regime in which the slip front lags behind the fluid pressure front (λ < 1, gray area) and the regime in which the slip front outpaces the fluid pressure front (λ > 1). (**B**) Scaled rupture speed as a function of the loading parameter *T*.

There are discrepancies between model and data that are due to the finite experimental system: In particular, at low pressure rates (i.e., large values of *T*), the slip front initiates in most of the cases after *L* already reached the opposite edge of the fault. For these cases, the corresponding λ values are not representative and are therefore biased because of the finite length of our experimental faults, which limit the propagation of both fronts in space (Fig. 4A).

In addition, we observe a strong correlation between the slip front propagation velocity $\dot{R}$ and the loading parameter *T* (Fig. 4B). Using our simplified model, we estimated $\dot{R}$ as a function of *T* throughout the entire injection duration. Our numerical simulations indicate that the rupture velocity is expected to slightly decrease over time (after an initial phase that depends on *T*) but tends to stabilize to nearly constant velocity at large time, consistent with our experimental observations (Fig. 3 and fig. S3). Furthermore, both our numerical and experimental results suggest that $\dot{R}$ scales with $T^{-m}$, with *m* ranging from −3/4 to −1/2 (Fig. 4B and fig. S6B). This scaling indicates that in critically stressed faults, slip fronts will nucleate earlier and propagate more rapidly as *T* decreases. This result is consistent with previous laboratory experiments, which showed that high fluid injection rates promote early nucleation of dynamic slip along faults in polymer (*37*) and rocks (*38*).

Aseismic slip propagating further than fluid pressure during injection was previously inferred indirectly from field data (*21*). Our results provide direct confirmation of this phenomenon in a crustal rock tested under upper crustal conditions (initial effective pressure equivalent to that at a depth of 2 to 3 km). Our experiments also show that rupture growth is primarily controlled by a loading parameter that combines information about the initial stress, fault strength, and injection rate, in agreement with simple models based on fracture mechanics (*21*, *22*). Under our tested conditions, the slip front does not propagate as $\sqrt{t}$, but rather at a nearly constant velocity, in agreement with our model predictions. More generally, fracture mechanics models (*22*, *39*) (see Materials and Methods) imply that the propagation rate of the slip front is indicative of the injection scenario (constant volume rate, pressure, or pressure rate), which may explain field studies reporting seismicity migration (natural or induced) that differ from the conventionally assumed square-root time dependence (*14*, *18*).

In nature, the occurrence of seismicity in response to fluid injection, even for small pressure change compared to hydrostatic values, indicates that faults are critically stressed, i.e., at a background stress close to the frictional strength (*40*, *41*), at least in intraplate regions. Therefore, we anticipate that the loading parameter *T* is small and faults should be prone to rapid propagation of aseismic slip beyond the pressurized region. However, in these cases, the predictive power of simple models based on constant friction and constant hydraulic properties might reach its limit, since strength perturbations due to rate-and-state effects (*35*, *36*) and variations in hydraulic properties due to stress changes and slip accumulation (*20*) can become substantial. Our experiment illustrate these limits: We only have approximate constraints on the value of *T*, and we detected time- and space-dependent variations in hydraulic diffusivity (fig. S4). More elaborate models that include some of these effects, such as rate-and-state friction, have been developed (*23*, *42*), but remain to be tested under realistic crustal conditions.

## MATERIALS AND METHODS

The material used was Lanhélin granite, a coarse-grained granite from Brittany, France. Intact Lanhélin granite has a very low permeability (lower than 10^−21^ m^2^, which is the resolution of our equipment) and is considered impermeable over the timescale of our experiments. A cylindrical sample of 40 mm in diameter was cored and precisely ground to a length of 100 mm. The cylinder was then saw cut at a 30° angle relative to its axis of revolution, creating an elliptical fault surface measuring 40 mm in width and 80 mm in length along strike. This surface was subsequently prepared using a surface grinder. Two 2-mm-diameter boreholes were drilled from both sides of the sample, passing through the material and intersecting the fault surface. The center of the boreholes were offset 4 to 5 mm from the sample’s edge to maximize the fluid diffusion path along the fault. The prepared, faulted sample was then enclosed in a Viton jacket and instrumented with eight strain gauges and three additional pore pressure transducers (*29*, *30*), as schematically shown in Fig. 1A. The pore pressure sensors have a contact diameter of 7 mm with the sample surface and were placed across the fault to ensure good hydraulic connection between the fault zone and the sensors dead volume. The instrumented sample was placed in the triaxial oil-medium apparatus at the Rock and Ice Physics Laboratory, University College London (*43*). The borehole at the upstream end of the sample was connected to a high-capacity servo-hydraulic pore fluid intensifier equipped with a pressure transducer and a linear variable differential transformer (LVDT) to monitor fluid volume changes. The borehole bottom end was sealed and connected to a closed reservoir with its own pressure transducer. Distilled water was used as the pore fluid.

Confining pressure and axial differential stress were independently controlled by an electromechanical pump and a servo-hydraulic actuator, respectively. Axial shortening was measured by external LVDTs, corrected for the stiffness of the loading column. Load was measured with an external load cell and corrected for seal friction. Differential stress was calculated as the corrected load divided by the sample’s cross-sectional area. Fault slip was estimated by projecting the corrected axial shortening onto the fault plane, and average normal and shear stresses on the fault were computed by resolving the triaxial stress state onto the inclined fault surface (*44*).

The pore pressure sensors were calibrated using the method detailed in (*30*), by imposing a set of constant, uniform confining and pore pressures and determining linear regression coefficients between output voltages and confining and pore pressure. From these coefficients, the local pore pressure at each sensor location was determined from the measured voltage and known confining pressure.

All mechanical data including load cell, pore pressure measurements, LVDT, and strain gauges were recorded during the entire experiments at a sampling rate of 1 Hz. In addition, the strain gauges, the pore pressure at the five locations and one external LVDT were recorded during each injection at a sampling rate of 20 kHz.

Experiments were conducted at a confining pressure of 80 MPa, with an initial pore pressure of 10 MPa. To determine the shear stress at fault slip onset ($\tau_{p}$) under constant pore pressure, an axial loading test was performed (fig. S1). The load was then reduced to a predetermined initial stress ($\tau_{0}$), after which the actuator position was held constant using the servo-controlled feedback loop based on external displacement sensors. This setup constituted a “stress relaxation” test (*20*, *24*, *45*), where elastic strain energy was stored in the loading column. Fault reactivation initiated the release of this stored energy through sample shortening via fault slip. Slip events were accompanied by a proportional reduction in applied stress, governed by the machine stiffness. This method ensured controlled fault slip, limiting runaway failure while still allowing for stick-slip behavior.

### Synthetic crack propagation using finite element modeling

To test the robustness of what we identified as the passage of the slip front on the strain gauges, we modeled the axial strain at the gauge locations that would arise from the propagation of a synthetic crack on the fault, following the finite element method (FEM) Green’s function approach presented in (*32*). Green’s functions relating slip on the fault and strain at gauge locations are first computed from the static equilibrium of one half of the sample, assuming the experimental boundary conditions (no displacement at the bottom, constant confining pressure of 70 MPa on lateral boundaries) and unit slip on the fault surface. Then, we consider a slip distribution δ corresponding to a circular crack growing from the injection site center with constant rupture speed $\dot{R}$ and stress drop Δτ. δ is convolved with Green’s functions to compute axial strains at gauges locations during the propagation of the simulated rupture. Our numerical results highlight that the first abrupt strain release recorded at the strain gauge location is consistent with the passage of the slip front (fig. S2).

### Inversion of the slip front velocity

For each injection, the first abrupt strain release recorded on each strain gauge was manually picked. The rupture initiation point and rupture velocity were then estimated using a least squares inversion method by comparing the experimental arrival times with theoretical arrival times calculated as follows.

The forward problem is modeled using a simplified assumption that the rupture front is circular. The theoretical arrival time at strain gauge *i*, denoted $t_{i}$, is given by

$$t_{i}=t_{0}+\frac{\sqrt{{\left(x-x_{i}\right)}^{2}+{\left(y-y_{i}\right)}^{2}}}{\dot{R}}$$ (2)

where $t_{0}$ is the rupture origin time, (x,y) are the coordinates of the rupture initiation point on the fault plane, $\dot{R}$ is the rupture velocity, and $\left(x_{i},y_{i}\right)$ are the coordinates of the strain gauge.

The inverse problem involves determining the model parameters *x*, *y*, $t_{0}$, and $\dot{R}$. To solve this, we used a grid-search approach, systematically exploring a range of possible values for each parameter and evaluating a likelihood function based on the least squares criterion

$$\underset{x,y,t_{0}}{\text{min}}\sum_{i=1}^{N}{\left(t_{i}^{\text{exp}}-t_{0}-\frac{\sqrt{{\left(x-x_{i}\right)}^{2}+{\left(y-y_{i}\right)}^{2}}}{\dot{R}}\right)}^{2}$$ (3)

where $t_{i}^{\text{exp}}$ are the experimentally measured arrival times and *N* is the total number of observations.

### Inversion of the fluid pressure along the fault

On the basis of the previous study (*46*), we developed an inversion procedure to estimate the time- and space-dependent hydraulic diffusivity D(x,t) along the experimental fault from sparse pressure observations. The two main elements are the parametrization of *D* and the way to update *D* to fit the observed data at the transducer locations. We first recall the forward problem (diffusion equation) and the objective function. We then discuss the parametrization and lastly provide an expression for the gradient, indicating how to iteratively update *D*.

### Forward modeling

The spatiotemporal evolution of fluid pressure *p* is modeled by the diffusion equation

$$\frac{\partial p}{\partial t}\left(x,t\right)=\nabla \cdot \left(D\left(x,t\right)\nabla p\right)$$ (4)

We applied Neumann condition at the domain boundary ($\partial_{n}p=0$) and Dirichlet condition at source locations [$p\left(x_{s},t\right)=p_{s}\left(t\right)$], where $x_{s}$ denotes the source positions, corresponding to the two boreholes along the fault (Fig. 1A), and $p_{s}$ denotes the imposed pressure. To account for dead-volume effects in the downstream pore pressure system, we constrained the inversion by imposing the fluid pressure at the injection site ($P_{1}$) as well as the pore pressure measured in the second borehole ($P_{5}$). The diffusion equation is solved with an explicit time-stepping scheme and a finite element method suited with a mesh description of the pressure.

### Objective function

We aim to find the optimal diffusivity field D(x,t), such that the simulated pressure $p\left(x_{r},t\right)$ (depending on *D*) matches the observed data $p^{\text{obs}}\left(x_{r},t\right)$ at pore pressure transducer locations $x_{r}$, by minimizing the least-squares misfit functional

$$J\left[D\left(x,t\right)\right]=\frac{1}{2}\iint \mathrm{dt}\;dx_{r}\;{\left|p\left(x_{r},t\right)-p^{\text{obs}}(x_{r},t)\right|}^{2}$$ (5)

Before explaining how to optimize the objective function, we first discuss the *D* parametrization.

### *D* parametrization

As only sparse pore pressure transducers are available, it is essential to constrain the inversion with a suitable parameterization for D(x,t). Instead of looking for values at each spatial positions x and time coordinates *t* that would not be well constrained by the data, the diffusivity field is parameterized using a spatial basis functions $B_{i}\left(x\right)$

$$D\left(x,t\right)=\sum_{i=1}^{N}D_{i}\left(t\right)B_{i}\left(x\right)$$ (6)

where $D_{i}\left(t\right)$ are the time-dependent weights to be inverted. On the basis of the acquisition geometry, we assume three independent regions (*N* = 3), each monitored by an individual fluid pressure transducer ($P_{2}$, $P_{3}$, and $P_{4}$), within which D(x,t) is assumed homogeneous in space. Thus $B_{i}\left(x\right)$ is simply 1 in region *i* and 0 in other regions.

The reconstruction from $D_{i}$ to D(x,t) is simply given by Eq. 6. The decomposition from D(x,t) to $D_{i}$ is obtained by minimizing the differences $\sum_{i=1}^{N}D_{i}(t)B_{i}\left(x\right)-D\left(x,t\right)$ in a least-squares sense. For each time *t*, it consists in solving a linear system

$$\sum_{i=1}^{N}{\langle B_{k},B_{i}\rangle }_{x}D_{i}\left(t\right)={\langle B_{k},D\left(x,t\right)\rangle }_{x}$$ (7)

where ${\langle ,\rangle }_{x}$ denotes the scalar product and *k* ranges from 1 to *N*.

Last, to allow for variations in diffusivity across several orders of magnitude, we define the logarithmic variable $D_{i}^{L}\left(t\right)=\text{log}\;\left(D_{i}\left(t\right)/D_{r}\right)$, so that $D_{i}\left(t\right)=D_{r}\;\text{exp}\;D_{i}^{L}\left(t\right)$. $D_{r}$ is a fixed diffusivity value. We thus aim at determining $D_{i}^{L}\left(t\right)$ for the three regions *i* and for all times *t*.

### Gradient derivation

The derivative of *J* with respect to $D_{i}^{L}\left(t\right)$ is given as

$$\frac{\partial J}{\partial D_{i}^{L}\left(t\right)}=\frac{\partial D_{i}\left(t\right)}{\partial D_{i}^{L}\left(t\right)}{\langle \frac{\partial D\left(x,t\right)}{\partial D_{i}\left(t\right)},\frac{\partial J}{\partial D\left(x,t\right)}\rangle }_{x}$$ (8)

By definition of $D_{i}^{L}\left(t\right)$, the first term reads $\partial D_{i}\left(t\right)/\partial D_{i}^{L}\left(t\right)=$ $D_{r}\;\text{exp}\;D_{i}^{L}\left(t\right)=D_{i}\left(t\right)$, while the second is simply $\partial D\left(x,t\right)/\partial D_{i}\left(t\right)=$ $B_{i}\left(x\right)$. The last term is more complex and can be obtained with the adjoint-state method (*47*), as

$$\frac{\partial J}{\partial D\left(x,t\right)}=\nabla p\left(x,t\right)\cdot \nabla \lambda_{p}\left(x,t\right)$$ (9)

where the adjoint pressure $\lambda_{p}\left(x,t\right)$ satisfies a similar diffusion equation as for the forward pressure p(x,t)

$$\frac{\partial \lambda_{p}}{\partial t}\left(x,t\right)=-\nabla \cdot \left(D\left(x,t\right)\nabla \lambda_{p}\right)-\int dx_{r}\delta \left(x-x_{r}\right)[p-p^{\text{obs}}\left(x_{r},t)\right]$$ (10)

The source term is zero, except for a spatial position at an observation transducer location. As for the forward pressure, zero-Neumann conditions are imposed to the adjoint pressure $\lambda_{p}$, but, here, $\lambda_{p}$ is set to 0 at the injection location. The adjoint diffusion equation is as stable as the forward diffusion equation, as it is solved backward, from the final time to the initial time. Combining the different expressions, the gradient reads

$$\frac{\partial J}{\partial D_{i}^{L}\left(t\right)}=D_{i}\left(t\right)\int dx\;B_{i}\left(x\right)\nabla p\left(x,t\right)\cdot \nabla \lambda_{p}\left(x,t\right)$$ (11)

The inversion is performed using a standard non-linear quasi-Newton (L-BFGS) algorithm. Results for diffusivity values and pore pressure fits are shown in fig. S4.

### Fracture mechanics model

Our experimental setup closely resembles the theoretical model explored in (*22*), whereby a planar frictional fault embedded in an infinite elastic medium is loaded by an initially uniform background stress $\tau_{0}$, and fluid injection is simulated by an on-fault pore pressure source at a point or in a small circular borehole of radius *a*. In our experiments, the sample is finite, but an infinite model should capture the early stages of slip nucleation and propagation. The loading parameter that controls the propagation of slip is given by

$$T=\left(1-\tau_{0}/f\sigma_{0}^{\prime }\right)/\left(\Delta p^{*}/\sigma_{0}^{\prime }\right)$$ (12)

where *f* is the friction coefficient on the fault (assumed constant), $\sigma_{0}^{\prime }$ is the effective normal stress applied on the fault, and $\Delta p^{*}$ is a characteristic fluid pressure related to the injection. Sáez *et al.* (*22*) treat two different injection scenarios: one where fluid is injected at constant volume rate, in which case $\Delta p^{*}$ is given by a combination of the injection rate, fault diffusivity and fault compressibility; and one where fluid is injected at constant pressure, in which case $\Delta p^{*}$ is simply the applied pore pressure at the borehole.

Here, we follow the steps of (*22*) and determine the solution for rupture size and fluid pressure in the case of injection at constant pressure rate, relevant to our experimental injection scenario.

The first step is to solve for the fluid pressure distribution along the the fault plane. Denoting p(r,t) the fluid pressure as a function of radial position *r* (measured from the borehole center) and time *t*, the radial diffusion problem is written as

$$\frac{\partial^{2}p}{\partial r^{2}}+\frac{1}{r}\frac{\partial p}{\partial r}=\frac{1}{\alpha }\frac{\partial^{2}p}{\partial t^{2}}$$ (13)

where α is the hydraulic diffusivity of the fault, assumed constant. The boundary condition at the well radius *a* is

$$p\left(a,t\right)=p_{0}+\mathrm{ct}$$ (14)

where $p_{0}$ is the initial (uniform) pore pressure and *c* is the constant pressurization rate. The solution is of the form

$$p\left(r,t\right)=p_{0}+\Delta p^{*}\Pi \left(r,t\right)$$ (15)

where $\Delta p^{*}={\mathrm{ct}}_{\text{diff}}$ is a characteristic pressure combining the injection rate *c* and the diffusion time around the well $t_{\text{diff}}=a^{2}/\alpha$ and Π(r,t) is a nondimensional function that characterizes the spatiotemporal pore pressure distribution. That function can be expressed as the product

$$\Pi \left(r,t\right)=\left(t/t_{\text{diff}}\right)\times F\left(r/a,t/t_{\text{diff}}\right)$$ (16)

where $F\left(r/a,t/t_{\text{diff}}\right)$ ranges from 1 at r=a to 0 at r→∞. In practice, we compute Π(r,t) by numerical inversion of the solution of (*13*) in the Laplace domain (*48*). The solution for p(r,t) provides us with the position *L*(*t*) of the fluid pressure front: Although there is no well-defined front in a diffusion problem, we define *L*(*t*) as the position *r* where pore pressure is $10^{-3}\Delta p^{*}$ above $p_{0}$. The factor 10^−3^ is arbitrary, but other definitions would offset *L*(*t*) by some value while retaining the same qualitative features.

We determine how a circular rupture expands in response to the pore pressure field p(r,t) using the fracture mechanics criterion *K* = 0, where *K* is the stress intensity factor at the rupture tip. This growth criterion assumes that the rupture is nonsingular, i.e., we neglect potential toughness terms arising from nonlinearities in the rupture cohesive zone. In terms of on-fault stresses, this criterion is expressed as (*22*)

$$\int_{0}^{R\left(t\right)}\frac{\Delta \tau \left(r,t\right)}{\sqrt{R{\left(t\right)}^{2}-r^{2}}}\mathrm{rdr}=0$$ (17)

where *R*(*t*) is the rupture radius and the stress drop is given by

$$\Delta \tau \left(r,t\right)=\tau_{0}-f\times [\sigma_{0}^{\prime }-\Delta p\left(r,t)\right]$$ (18)

Note that $\Delta p\left(r,t\right)=p\left(r,t\right)-p_{0}$ is equal to *ct* inside the well (r≤a). It is therefore obvious that there will be no rupture at all unless

$$\mathrm{ct}\geq \sigma_{0}^{\prime }-\tau_{0}/f$$ (19)

Using Eq. 16, Eq. 17 is rewritten as

$$\frac{1}{\bar{R}}\int_{0}^{\bar{R}}\frac{F\left(r,t\right)r}{\sqrt{{\bar{R}}^{2}-r^{2}}}\mathrm{dr}=T/\bar{t}$$ (20)

where we have defined normalized quantities $\bar{R}=R/a$ and $\bar{t}=\mathrm{t\alpha}/a^{2}$, and the loading parameter

$$T=\frac{1-\tau_{0}/\left(f\sigma_{0}^{\prime }\right)}{\Delta p^{*}/\sigma_{0}^{\prime }}$$ (21)

where again we recall that $\Delta p^{*}={\mathrm{ca}}^{2}/\alpha$. With this notation, the rupture initiation condition is expressed as

$$\bar{t}\geq T$$ (22)

We solve numerically for *R* in Eq. 20 as a function of *T* and time *t*. The results are shown in fig. S5A, where we also show the fluid front position. For small *T* (large injection rate, dark red lines), the rupture front significantly outpaces the fluid pressure front. For large *T* (small injection rate, light red lines), the rupture front initiates only when sufficient pressure has been reached at the well (Eq. 22) and is initially behind the fluid pressure front.

By contrast with injection at constant volume rate, here, the amplification factor λ=R/L depends on both *T* and time (fig. S5B). In all cases, λ increases with increasing time. For large values of *T* (marginally pressurized faults, small injection rate), rupture initiates when *L* is already large, so λ is initially small (rupture front lagging behind fluid front). With increasing time, λ gradually increased and *R* eventually outpaces *L*. For small values of *T* (critically stress fault, large injection rate), *R* rapidly outgrows *L*.

The rupture growth velocity can be extracted from the model (fig. S6). Beyond the rupture initiation phase, for $\bar{t}$ much larger than *T*, the rupture velocity tends to decrease slightly with increasing time, whereas the fluid front velocity decreases more rapidly. There is a clear trend of larger rupture velocities at lower *T*, i.e., faster injection rates and/or higher background stress promote faster rupture growth (fig. S6B). Rupture velocity is found to scale with the loading parameter as *T*^−1/2^ to *T*^−3/4^, depending on injection time.
