# Supplementary material for: Propagation of fluid-driven aseismic slip fronts under upper crustal conditions
Source: Sci Adv. 2026 Jul 8;12(28):eaeb0234. doi: 10.1126/sciadv.aeb0234 (PMC13344294; doi:10.1126/sciadv.aeb0234)
Supplement: Supplementary file 1 [file sciadv.aeb0234_sm.pdf]

Science Advances
AAAS

Supplementary Materials for

Propagation of fluid-driven aseismic slip fronts under upper crustal conditions

François Passelègue *et al.*

Corresponding author: François Passelègue, francois.passelegue@cnrs.fr

Sci. Adv. **12**, eaeb0234 (2026)
DOI: 10.1126/sciadv.aeb0234

This PDF file includes:

Table S1
Figs. S1 to S6

Experiment	τ_0/τ_p [MPa]	P_c [MPa]	P_f [MPa]	c [MPa/sec]
phase 1	0.6	80	10	0.15
phase 2	0.6	80	10	1.5
phase 3	0.6	80	10	15
phase 4	0.9	80	10	0.015
phase 5	0.9	80	10	0.15
phase 6	0.9	80	10	1.5
phase 7	0.9	80	10	15

Table S1: Summary of the initial state of stress and of the pressure rate imposed during the seven injections conducted in this study.

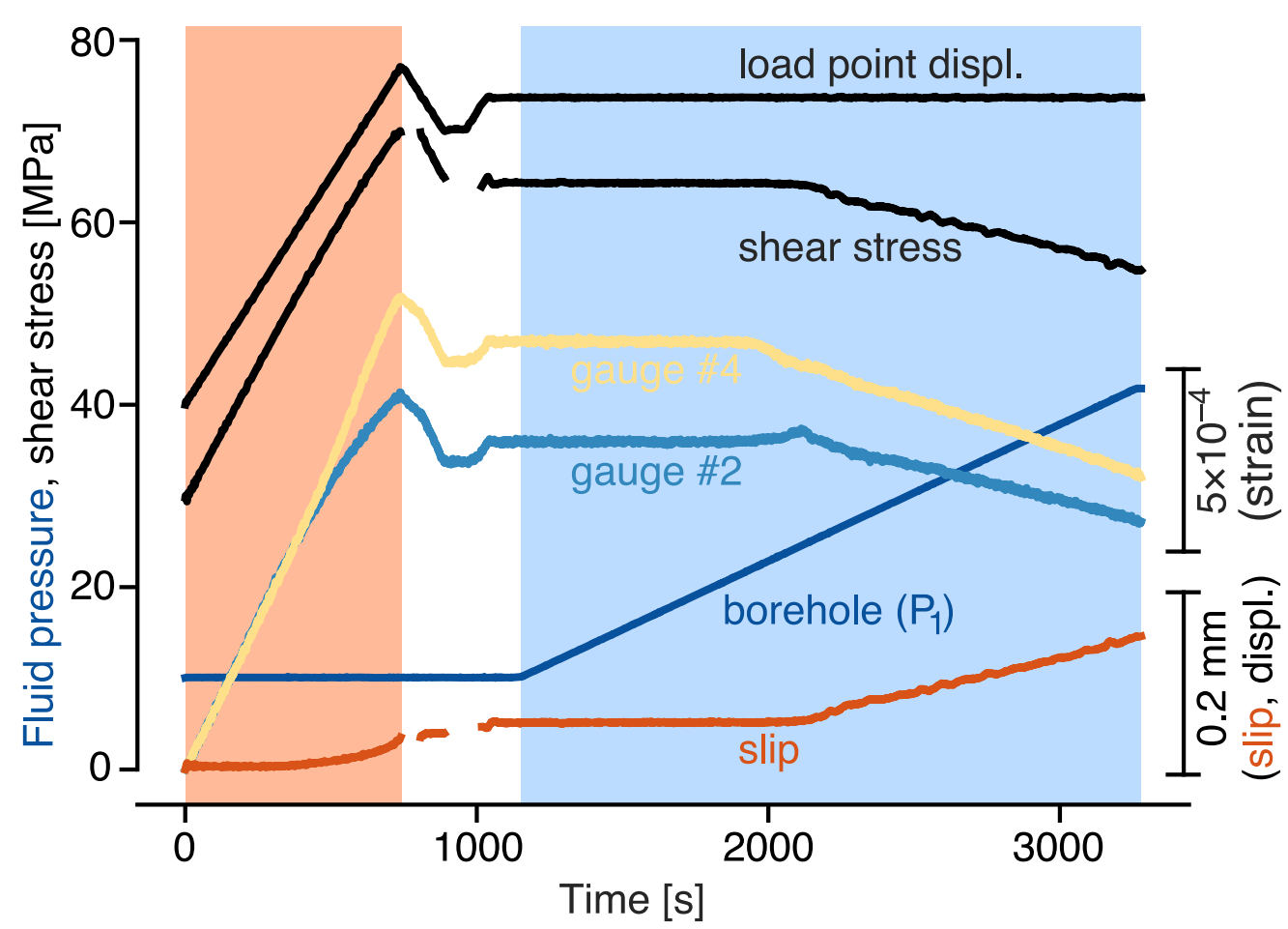

Figure S1: Experimental protocol used in the study. Temporal evolution of shear stress, load-point displacement, fluid pressure in the injection borehole (blue solid line), strain measured by two gauges, and fault slip. The red-shaded region corresponds to the loading phase used to probe the fault frictional strength, followed by an unloading and reloading phase to the target shear stress (maintained with constant load point displacement of the piston). The blue-shaded region represents fluid injection at a constant pressure rate (c = 0.015 MPa/s). The gaps in the shear stress and slip curves correspond to a "dead zone" associated with piston friction, during which the external load measurements are not reliable (*49*) and therefore not reported. As slip is derived from external load measurements, it is similarly affected. We used load and displacement data only during intervals when the piston was moving in a single direction.

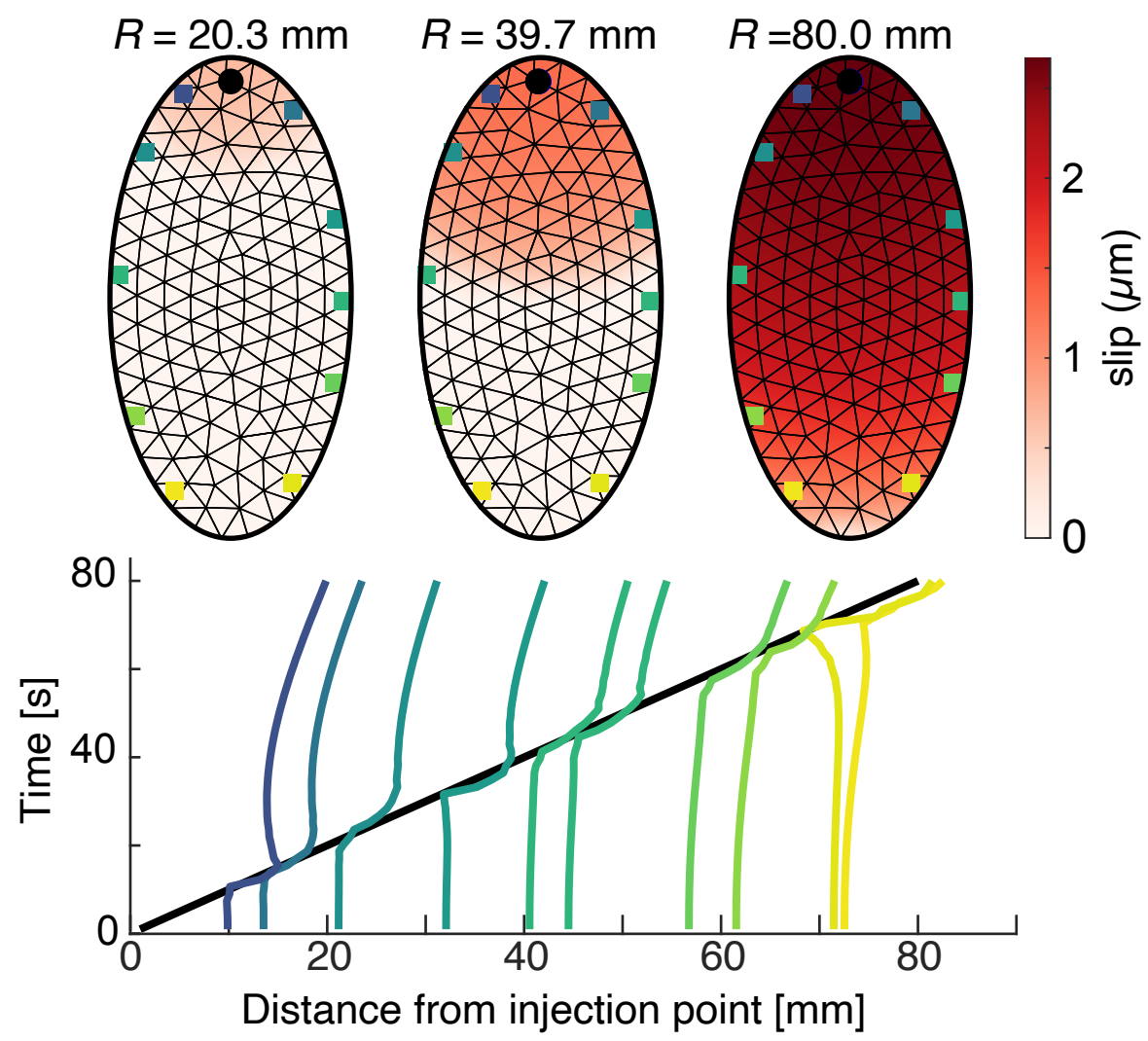

Figure S2: Illustration of the synthetic rupture model with a circular expanding slip patch at constant rupture speed. Top: three snapshots of slip distribution for three rupture dimensions. Colored squares indicate positions where axial strain is computed from synthetic Green's functions. Bottom: Simulated axial strain records, plotted as a function of distance. from injection point (i.e., distance from rupture initiation point). The onset of strain decrease is a good indicator of the position of the slip front (black line).

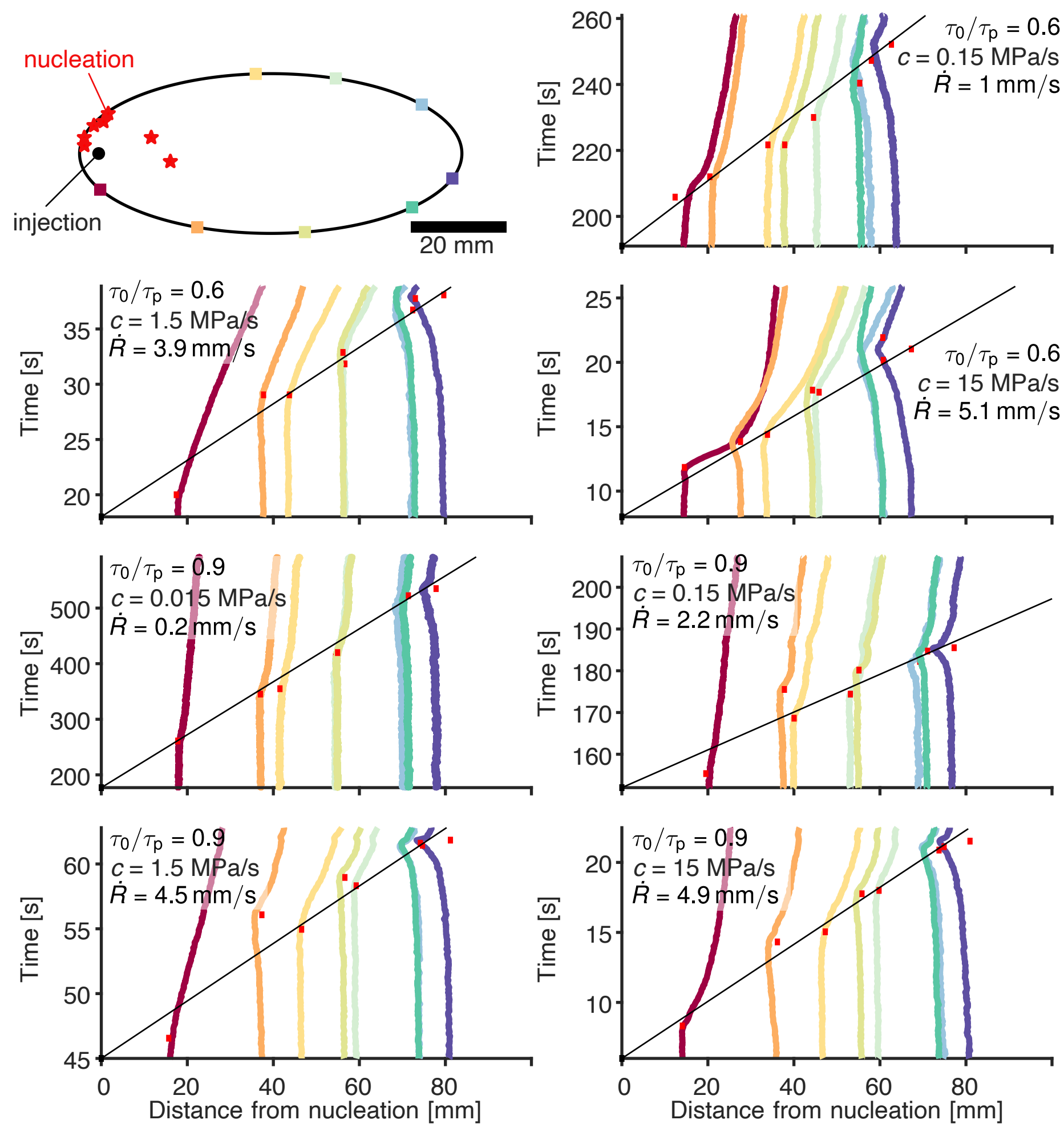

Figure S3: Inversion of rupture-front velocity for all injection events. The first panel shows the inferred rupture initiation points and the locations of strain gauges (indicated by colored squares). The subsequent panels present the temporal evolution of strain recorded by each strain gauge positioned around the fault. The onset of strain drop at each gauge (marked by red squares) was used to invert for the rupture-front velocity. The best-fit rupture velocity, $\dot{R}$, is represented by the solid black line.

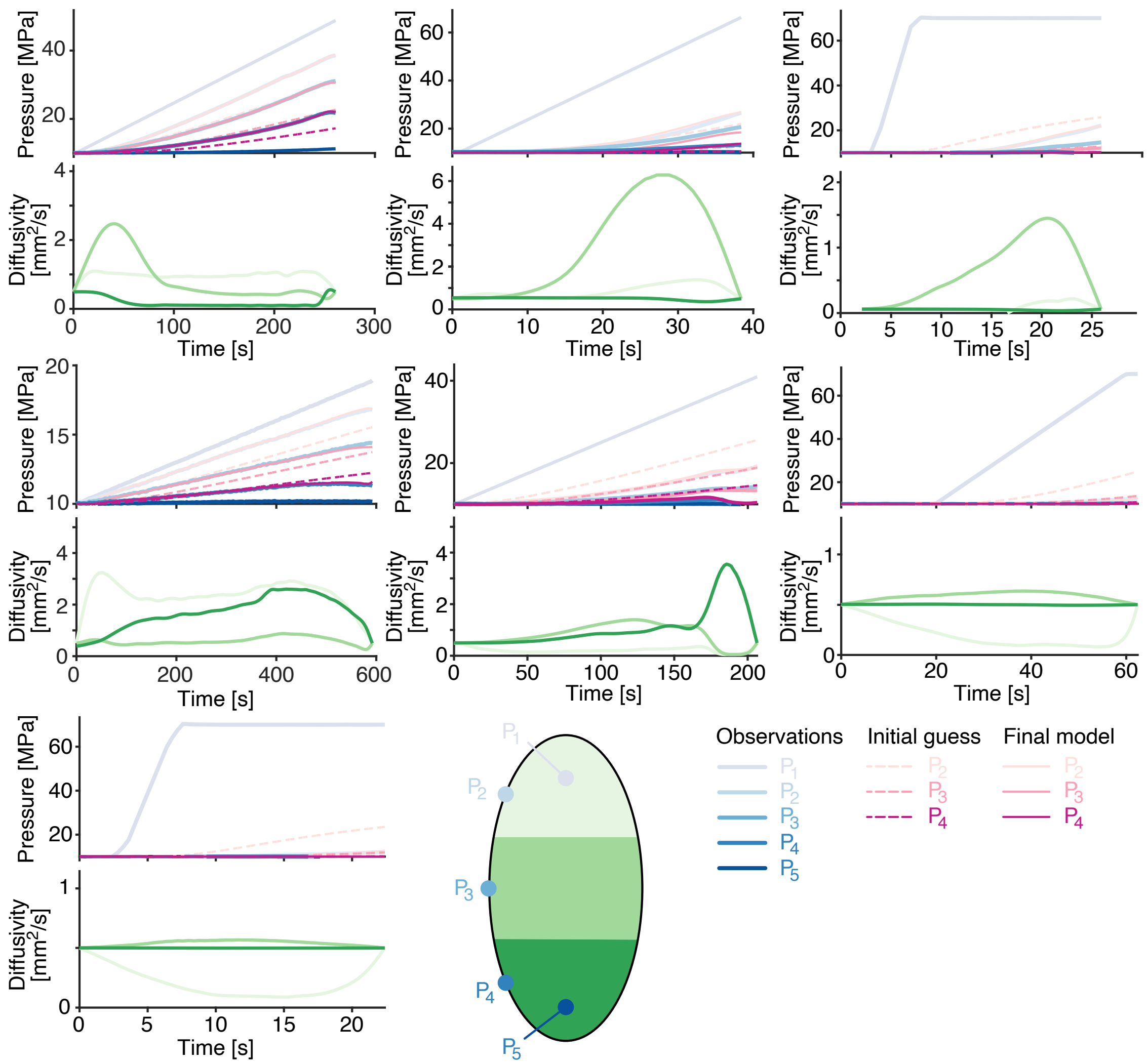

Figure S4: Inversion of fault zone hydraulic diffusivity. Each pair of subplots displays the measured pore pressure data (blue lines), the initial model predictions assuming uniform diffusivity (dashed purple lines), and the final model predictions (solid purple lines) using inverted diffusivities for each fault regions (green curves). Experimental conditions are summarized in Table S1. The bottom-left panels show the locations of the pore pressure transducers and the corresponding fault regions over which hydraulic diffusivity was inverted as a function of time. Colored regions indicate the spatial domains associated with each transducer, corresponding to the curves shown in the adjacent subplots.

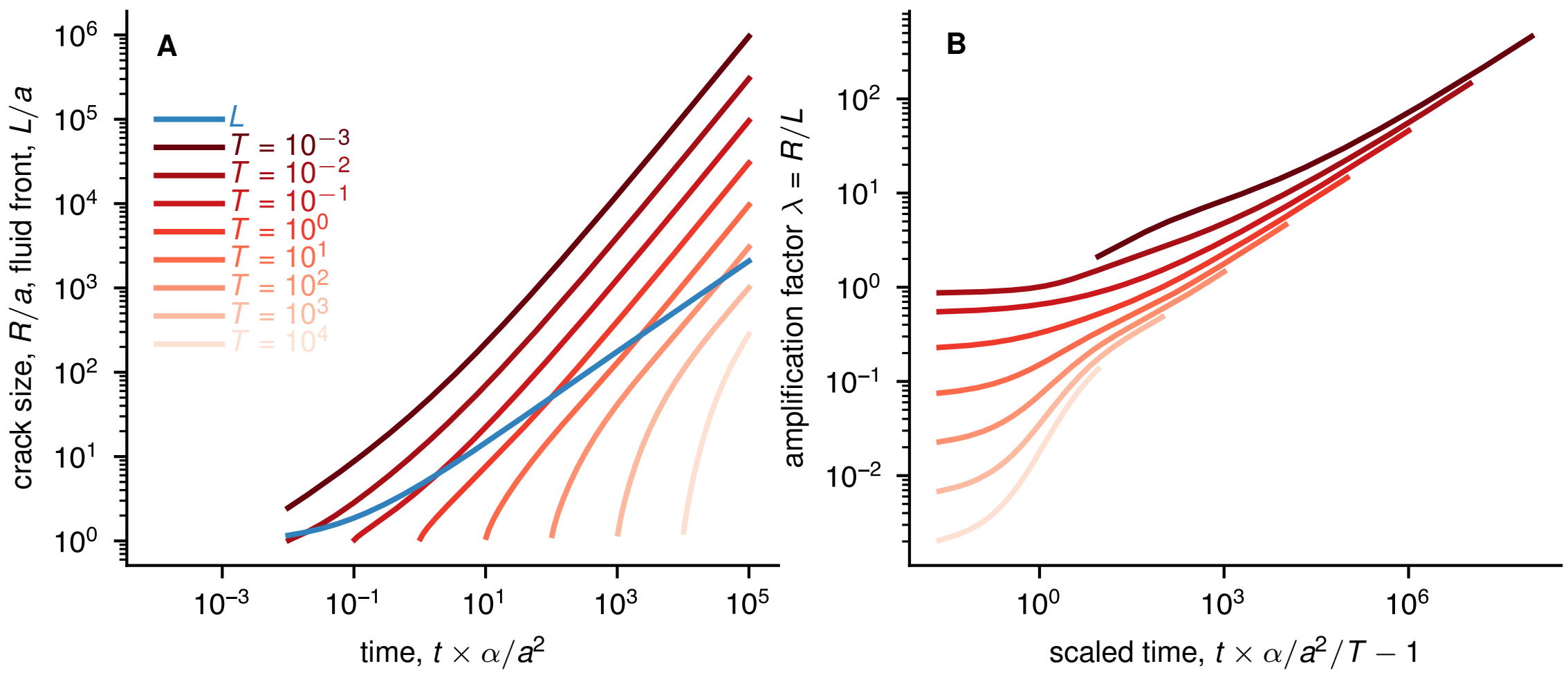

Figure S5: Solution of rupture and fluid diffusion model for constant pressurisation rate (Equation 20). (**A**) Rupture front (red) and fluid front (blue) expansion as a function of normalised time, for a range of loading parameters T. Note that rupture initiates when $t\alpha/a^2$ becomes larger than T. (**B**) Amplification factor $\lambda = R/L$ as a function of rescaled time $t\alpha/a^2/T - 1$.

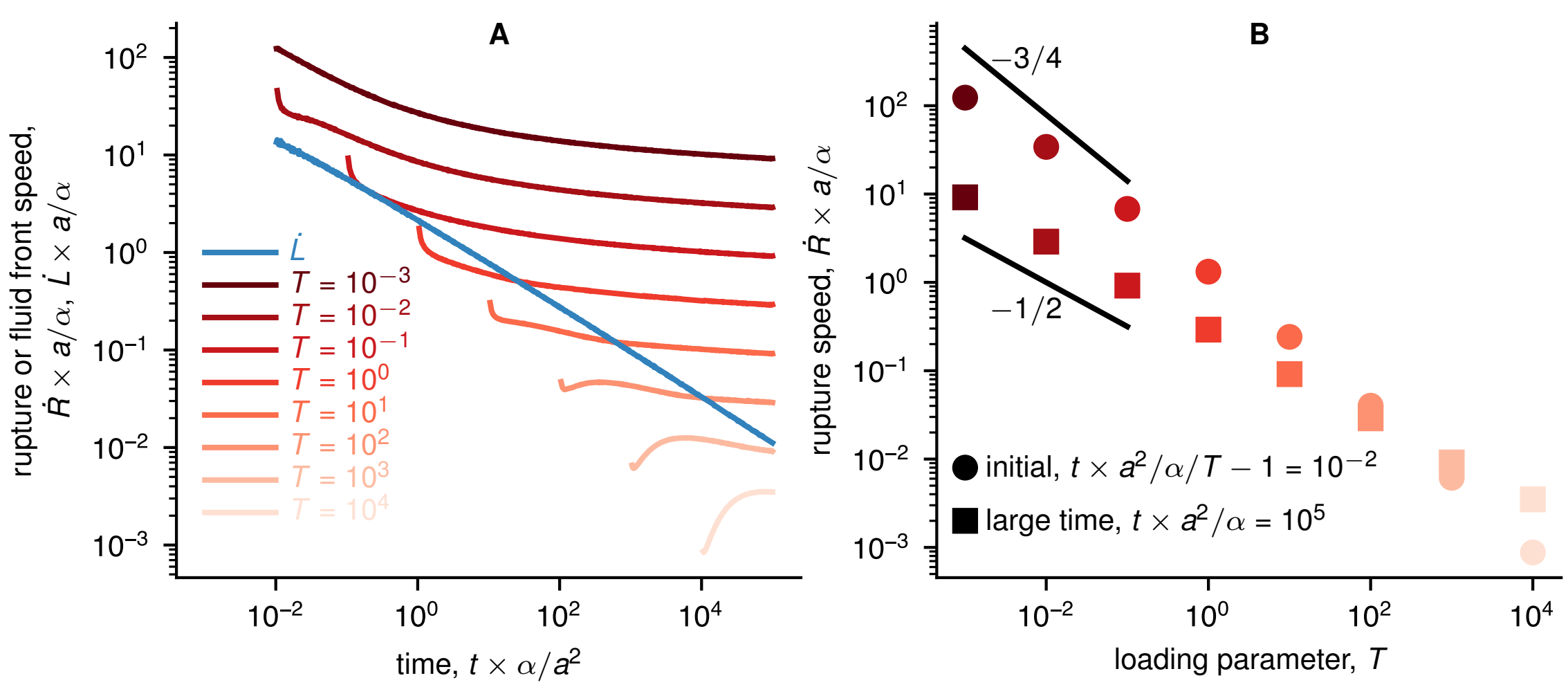

Figure S6: Rupture and fluid migration speeds. (**A**) Rate of rupture (red) and fluid front (blue) propagation as a function of time. (**B**) Initial rupture speed (circles) and rupture speed at large time (squares) as a function of loading parameter.
